# Multitargeted antibacterial mechanisms of Iturin A derived from *Bacillus amyloliquefaciens* against tomato bacterial wilt

**DOI:** 10.3389/fmicb.2026.1944894

**Published:** 2026-10-20

**Authors:** Jinyu Yang, Tianbo Liu, Zhanjie Li, Tiansheng Zhu, Luping Chi, Xu Yan, Baoxiang Pan, Jinchang Liang, Xiaoqiang Wang

**Affiliations:** 1 State Tobacco Monopoly Administration (China National Tobacco Corporation), Key Laboratory of Tobacco Pest Monitoring Controlling & Integrated Management, Tobacco Research Institute of Chinese Academy of Agricultural Sciences, Qingdao, China; 2 Hunan Tobacco Research Institute, Changsha, China; 3 Enshi Tobacco Science and Technology Center, Enshi, China; 4 College of Agriculture, Tarim University, Alar, China

**Keywords:** antibacterial activity, cell membrane, electron transport chain, Iturin A, *Ralstonia solanacearum*

## Abstract

Tomato bacterial wilt is a devastating disease caused by *Ralstonia solanacearum*. This study aimed to evaluate the antibacterial activity of Iturin A, a cyclic lipopeptide produced by *Bacillus amyloliquefaciens* strain Cas02, against *R. solanacearum* and investigate its mechanisms of action. The results indicated that Iturin A effectively inhibited the growth of *R. solanacearum* with an MIC and IC_50_ of 25.00 and 15.79 μg/mL, respectively. In pot experiments, Iturin A significantly reduced disease incidence with a control efficacy of 74.27%. Microscopy observations revealed that Iturin A induced severe morphological damage, including membrane disruption and cytoplasmic leakage, and inhibited biofilm formation. Additionally, Iturin A caused the accumulation of intracellular reactive oxygen species, a decrease in succinate dehydrogenase activity, reductions in NAD^+^ and NADH content, and alterations in cytochrome c oxidase staining, indicating disruption of the electron transport chain and energy metabolism. Transcriptome analysis further indicated the downregulation of genes related to the membrane structure and type III secretion system. Overall, these findings suggest that Iturin A may exert antibacterial effects through multiple pathways, including impairment on cell integrity, biofilm formation, energy metabolism, and virulence-associated functions. This study provides a basis for further exploration of Iturin A as a potential antibacterial agent for managing tomato bacterial wilt.

## 1 Introduction

Tomato (*Solanum lycopersicum* L.) is one of the most widely cultivated vegetable crops worldwide (Meshram and Adhikari, 2024), valued for its genetic diversity, rich nutritional profile, and versatile applications, across the agricultural and food industries (Domínguez et al., 2025; Li J. et al., 2024; Martí et al., 2016). However, tomato production is severely threatened by pathogenic infections throughout the growth cycle (Bahramisharif and Rose, 2019). Among these, bacterial wilt caused by *Ralstonia solanacearum* is one of the most devastating soil-borne diseases in tomato-producing regions, resulting in substantial yield and quality losses (Sun et al., 2023). *Ralstonia solanacearum* deploys multiple virulence determinants, including type III secretion system (T3SS) effectors, exopolysaccharides (EPS), and cell-wall-degrading enzymes secreted via the type II secretion system (Peeters et al., 2013). The pathogen invades through root wounds, colonizes the xylem, and produces copious extracellular polymeric substances that occlude vascular xylem, thereby disrupting water transport and causing rapid wilting and plant death (Zhu et al., 2025; Genin and Denny, 2012). Although chemical bactericides remain the predominant control measure owing to their efficacy and convenience, growing concerns over environmental residues and resistance development have highlighted the need for alternative antibacterial agents.

Iturin A, a cyclic lipopeptide produced by *Bacillus* spp., has attracted considerable attention as a potential antibacterial agent (Wan et al., 2022). Structurally, it comprises a β-hydroxy fatty acid chain linked to a cyclic heptapeptide, forming a characteristic amphiphilic architecture, and occurs naturally as eight isomers (A1–A8) (Zhang D. et al., 2025). Iturin A exhibits broad-spectrum antifungal activity; for instance, it suppresses pathogenic *Fusarium* species associated with wheat scabs by targeting cell-membrane components (Yuan et al., 2025) and inhibits *Aspergillus niger* induced fruit rot (Zhang et al., 2024). These properties have facilitated its application in both food preservation and agricultural disease management (Ambrico and Trupo, 2017; Malik et al., 2024; Xie et al., 2023; Ye et al., 2012). At the molecular level, its antifungal action is primarily attributed to disruption of fungal cell structures and interference with cellular metabolism (Zhang D. et al., 2025). Beyond antifungal activity, Iturin A has also demonstrated antibacterial effects against *Paenibacillus larvae* (Benitez et al., 2012), *Staphylococcus aureus* (Rasiya and Sebastian, 2021), and *Salmonella typhimurium* (Yaraguppi et al., 2022). Moreover, although Iturin A has been reported to exhibit pronounced inhibitory activity against *R. solanacearum* (Cao et al., 2018; Chen et al., 2019), a comprehensive understanding of its antibacterial mechanism remains lacking, and its antibacterial spectrum has not been fully characterized, which limits its practical application.

In our previous work, we isolated and screened a strain of *Bacillus amyloliquefaciens* Cas02, which exhibited significant antibacterial activity against *R. solanacearum*, and identified Iturin A as the principal antibacterial component. Building on this work, the present study used purified Iturin A to systematically evaluate its antibacterial activity, investigate its multitargeted mechanism of action and validate its biocontrol efficacy against tomato bacterial wilt. This work expands the known bioactivity profile of Iturin A and provides a theoretical basis for the development of lipopeptide-based biopesticides for the management of bacterial wilt.

## 2 Materials and methods

### 2.1 Materials and reagents

Iturin A (CAS No. 52229-90-0) was obtained from Sigma-Aldrich. Kasugamycin (2% active ingredient) was obtained from Hokko Chemical Industry Co., Ltd. (Japan). *Bacillus amyloliquefaciens* Cas02, *R. solanacearum* Cas800, *Pseudomonas syringae* Cas23, *Pectobacterium aroidearum* JM09, and *Pectobacterium carotovorum* Cas20 were maintained in our laboratory culture collection.

### 2.2 Extraction and purification of Iturin A

*Bacillus amyloliquefaciens* Cas02 was cultured in liquid Luria–Bertani (LB) medium at 28 °C and 200 rpm for 5 days. The cell-free supernatant was collected by centrifugation and mixed with macroporous resin at room temperature for 4 h to adsorb the fermentation products. The resin was packed into a glass chromatographic column and washed sequentially with deionised water and 40% (v/v) aqueous methanol to remove impurities. The target fraction was then eluted with 1 L of anhydrous methanol. The eluate was concentrated using a rotary evaporator and subsequently lyophilised to obtain crude powder. For further purification, the crude powder was redissolved in methanol and subjected to preparative high-performance liquid chromatography (prep-HPLC) with a mobile phase of acetonitrile/water (40:60, v/v) at a flow rate of 48 mL/min, monitored at 220 and 280 nm. The target fraction was collected, concentrated by rotary evaporation to remove acetonitrile, and lyophilised to yield high-purity Iturin A (Chu et al., 2021).

### 2.3 Antibacterial activity of Iturin A

#### 2.3.1 Disc-diffusion assay

The antibacterial spectrum of Iturin A was assessed against four plant pathogenic bacteria―*R. solanacearum*, *P. syringae*, *P. aroidearum*, and *P. carotovorum*―using a disc-diffusion method described by Wang et al. (2016). Each pathogen was cultured in liquid nutrient broth (NB) at 28 °C for 24 h. Cells were harvested, resuspended in sterile water, and adjusted to an OD_600_ of 0.4. Sterile filter-paper discs were loaded with 10 μL of Iturin A (20 mg/mL), air-dried, and placed on NB agar plates; discs loaded with sterile water served as negative controls. Each plate was then spray-inoculated with 300 μL of the respective bacterial suspension using an aerosol sprayer and incubated at 28 °C for 24 h. Antibacterial activity was determined by the presence of inhibition zones. All assays were performed in triplicate.

#### 2.3.2 Determination of minimum inhibitory concentration and half-maximal inhibitory concentration against *Ralstonia solanacearum*

The minimum inhibitory concentration (MIC) of Iturin A was determined by broth microdilution following the method of Chi et al. (2023), Wiegand et al. (2008). *Ralstonia solanacearum* Cas800 was cultured in LB medium at 28 °C for 24 h, harvested, and adjusted to an OD_600_ of 0.4. Aliquots of the bacterial suspension (10 μL) were inoculated into 190 μL of fresh LB medium supplemented with Iturin A at final concentrations of 0, 1, 2, 5, 10, 20, 25, and 30 μg/mL. Uninoculated LB medium (0 μg/mL Iturin A) served as the blank control. After incubation at 28 °C for 24 h, the MIC was defined as the lowest concentration producing ≥ 99% growth inhibition relative to the untreated control (Zhao et al., 2025). For the determination of the half-maximal inhibitory concentration (IC₅₀), *R. solanacearum* Cas800 (OD_600_ = 0.4) was inoculated at 5% (v/v) into LB medium containing Iturin A at 5, 15, 20, 25, 30, 40, and 80 μg/mL. After 24 h of incubation, OD_600_ values were recorded, and the inhibition rate was calculated as follows:


$$\text{Inhibition rate}\;(\%)=1-\frac{{{\mathrm{OD}}_{600}}^{\text{treated}}-{{\mathrm{OD}}_{600}}^{\text{blank}}}{{{\mathrm{OD}}_{600}}^{\text{control}}-{{\mathrm{OD}}_{600}}^{\text{blank}}}\times 100$$

The IC₅₀ was obtained by non-linear regression analysis. Both MIC and IC₅₀ assays were conducted in triplicate.

#### 2.3.3 Pot experiments for biocontrol efficacy

Pot experiments comprised three groups: a negative control (CK, sterile water), a positive control (kasugamycin), and a treatment group (Iturin A). Each group contained 20 tomato seedlings (cv. Guifei) at the four-true-leaf stage, transplanted 10 days prior. Each pot (top diameter 8 cm, height 7 cm, bottom diameter 6 cm) contained about 65 g of soil. Soil moisture was maintained at approximately 60% field water capacity throughout the experiment. Plants were maintained under a 16 h light/8 h dark photoperiod at 26 °C and 60% relative humidity. Kasugamycin or Iturin A (20 mL at 100 μg/mL) was irrigated into the soil of each plant; the same volume of sterile water was applied as a soil drench to each plant; the CK group received an equal volume of sterile water. After 2 days, each seedling was challenge-inoculated by soil drenching with 20 mL of *R. solanacearum* Cas800 suspension (OD_600_ = 0.4). Disease severity was scored on a 0–5 scale beginning 7 days post-inoculation (Lee et al., 2020). On day 15, disease index (Liang et al., 2025) and control efficacy (Meng et al., 2024) were calculated as follows:


$$\text{Disease incidence}\;(\%)=\frac{\text{Number of diseased plants}}{\text{Total number of plants}}\times 100$$

$$\begin{array}{l} \text{Disease index}(\%)\\ =\frac{\sum (\begin{array}{l} \text{number of plants}\;\mathrm{at}\;\text{each severity level}\\ \times \text{corresponding severity level} \end{array})}{\left(\begin{array}{l} \text{total number of plants}\\ \times \text{highest severity level} \end{array}\right)}\times 100 \end{array}$$

$$\begin{array}{l} \text{Control efficacy}\;(\%)\\ =\frac{\text{disease index}\_\mathrm{CK}-\text{disease index}\_\text{treated}}{\text{disease index}\_\mathrm{CK}}\times 100 \end{array}$$

### 2.4 Effects of Iturin A on cell structure and physiology of *Ralstonia solanacearum*

#### 2.4.1 Morphological and ultrastructural observations

*Ralstonia solanacearum* Cas800 cultured overnight was harvested by centrifugation, washed three times with phosphate-buffered saline (1 × PBS), and resuspended in PBS containing Iturin A at 0 (control), 15, 25, or 50 μg/mL. After incubation at 28 °C for 5 h, cells were collected (5,000 × g, 4 °C, 10 min), rinsed twice with PBS, and fixed with 2.5% glutaraldehyde for 2 h at room temperature. For scanning electron microscopy (SEM), fixed cells were dehydrated through a graded ethanol series, sputter-coated with gold, and observed on a Hitachi SEM (Hitachi, Japan) at an accelerating voltage of 20 kV.

For transmission electron microscopy (TEM), fixed cells were processed according to Shi et al. (2024). Ultrathin sections (60–80 nm) were prepared with an ultramicrotome, double-stained with uranyl acetate (50% ethanol-saturated) and lead citrate (15 min each), and examined on a Hitachi TEM (Hitachi, Japan).

#### 2.4.2 Cell-membrane integrity

The effect of Iturin A on cell membrane integrity was assessed using a green fluorescent protein (GFP)-expressing *R. solanacearum* constructed following the protocol of Chu et al. (2021). The recombinant strain was cultured in LB medium at 28 °C for 24 h and adjusted to an OD_600_ of 0.1. Iturin A was added to the final concentrations of 0 (control),16, 25, and 50 μg/mL. Aliquots (200 μL) were dispensed into 96-well plates and incubated at 28 °C for 24 h. GFP fluorescence of intact cells was measured using a microplate reader (Tecan, Switzerland) at excitation/emission wavelengths of 488/515 nm.

#### 2.4.3 Biofilm formation

Biofilm formation was quantified by crystal-violet staining as described by Stepanović et al. (2000). *Ralstonia solanacearum* Cas800 was cultured in LB medium for 12 h and diluted to an OD_600_ of 0.4. The diluted culture was inoculated into fresh LB medium at 1:30 ratio, and 100 μL aliquots were transferred into a 96-well plate. Iturin A was added to achieve final concentrations of 0, 10, 16, and 25 μg/mL. After static incubation at 28 °C for 72 h, adherent biofilms were stained with 2% (w/v) crystal violet and quantified by measuring the absorbance at 570 nm using a microplate reader (Tecan, Switzerland). All measurements were performed in triplicate.

#### 2.4.4 Reactive oxygen species accumulation

Intracellular reactive oxygen species (ROS) levels were quantified using a 2′,7′-dichlorodihydrofluorescein diacetate (DCFH-DA) probe (Beyotime, China). *Ralstonia solanacearum* Cas800 (OD_600_ = 0.4) was treated with Iturin A at 0, 16, 25, and 50 μg/mL at room temperature for 5 h. Cells were washed twice with 1 × PBS and incubated with DCFH-DA at room temperature for 4 h. After two additional PBS washes, cells were resuspended in PBS and fluorescence intensity was recorded using a microplate reader (Tecan, Switzerland) at excitation/emission wavelengths of 488/525 nm.

#### 2.4.5 Electron transport chain (ETC) activity

To evaluate the effect of Iturin A on the ETC of *R. solanacearum*, succinate dehydrogenase (SDH) activity, cytochrome c oxidase (CcO) activity, and NAD^+^/NADH levels were determined. *Ralstonia solanacearum* Cas800 (OD_600_ = 0.4) was harvested, washed twice with 1 × PBS, and treated with Iturin A at 16, 25, and 50 μg/mL at room temperature for 5 h. After two additional washes, cells were collected for analysis. SDH activity was measured using an SDH Activity Assay Kit (Solarbio, Beijing, China). NAD^+^/NADH ratios and CcO activity were determined using an NAD^+^/NADH Assay Kit with WST-8 and a Cytochrome c Oxidase Staining Kit with DAB (3,3-Diaminobenzidine), respectively (Beyotime, China). All assays were performed in triplicate.

### 2.5 Transcriptome sequencing and analysis

*Ralstonia solanacearum* Cas 800 cells treated with 16 μg/mL Iturin A for 24 h and untreated controls (three biological replicates per group) were harvested for total RNA extraction using a VeZol-Pure Total RNA Isolation Kit (Vazyme Biotech, China). RNA integrity and concentration were assessed using an Agilent 2100 Bioanalyzer (Agilent Technologies, USA) and a NanoPhotometer^®^ spectrophotometer (Implen, Germany), respectively. cDNA libraries were constructed using a TransScript One-Step gDNA Removal and cDNA Synthesis SuperMix (Vazyme Biotech, China) and sequenced on an Illumina NovaSeq 6000 platform (Novogene, China). Differentially expressed genes (DEGs) were identified using DESeq2 with cutoff criteria of |log₂ fold change| > 1 and adjusted *p*-value < 0.05. Functional and pathway enrichment analyses of DEGs were performed using the Gene Ontology (GO) and Kyoto Encyclopedia of Genes and Genomes (KEGG) databases.

### 2.6 Quantitative real-time PCR validation

Total RNA was extracted from *R. solanacearum* Cas800 treated with 16 μg/mL Iturin A for 24 h and from untreated controls as described in Section 2.5. cDNA was synthesised, and quantitative real-time PCR (qRT-PCR) was performed using SYBR Green qPCR Master Mix (Vazyme Biotech, China). Gene-specific primers (Supplementary Table S1) were designed based on the *R. solanacearum* Cas800 genome and synthesised by Qingdao Weilai Biotechnology Co., Ltd. The cell-division gene *ftsZ* served as the internal reference for normalisation. Relative gene expression was calculated using the 2^−ΔΔCt^ method (Zhang S. et al., 2025).

### 2.7 Statistical analysis

All experiments were performed with three technical replicates and three independent biological replicates. Data were subjected to one-way ANOVA under a completely randomized design, followed by Duncan’s multiple range test (DMRT) for mean separation at *p* < 0.05, using R software.

## 3 Results

### 3.1 Antibacterial activity of Iturin A against *Ralstonia solanacearum*

The purity and composition of the extracted Iturin A were verified by HPLC (Figure 1A). The purity of the prepared Iturin A was approximately 90%. The purified compound exhibited three peaks corresponding to Iturin A2, A3, and A4 (Figure 1B), with a peak area ratio of 4.9:1.2:1―consistent with the Iturin A standard (Chu et al., 2021).

**Figure 1:**
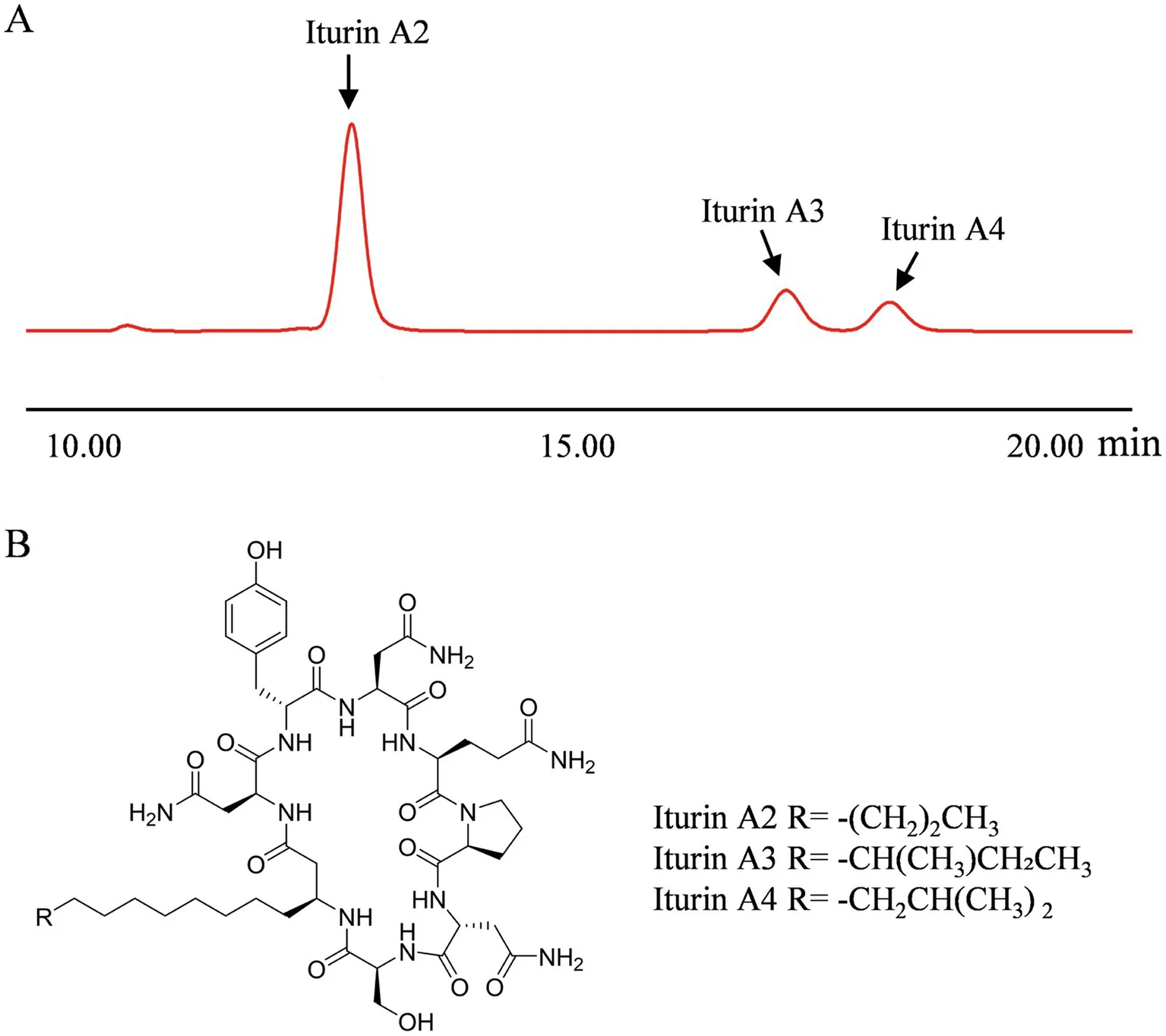
Purity and composition of extracted Iturin A. **(A)** High-performance liquid chromatography detection peak chromatogram of the extracted Iturin A sample. **(B)** Structural diagram of Iturin A used in this study.

The antibacterial spectrum of Iturin A was evaluated against four plant pathogenic bacteria (Figure 2A). At a concentration of 20 mg/mL, Iturin A displayed pronounced antibacterial activity against *R. solanacearum*, with an inhibition zone of 26 mm, whereas only weak inhibition (inhibition zone ≤10 mm) was observed against *P. syringae*, *P. aroidearum*, and *P. carotovorum* (Figure 2A). The MIC and IC_50_ of Iturin A against *R. solanacearum* were determined by broth microdilution, were 25.00 μg/mL (Figure 2B) and 15.79 μg/mL (Figure 2C), respectively.

**Figure 2:**
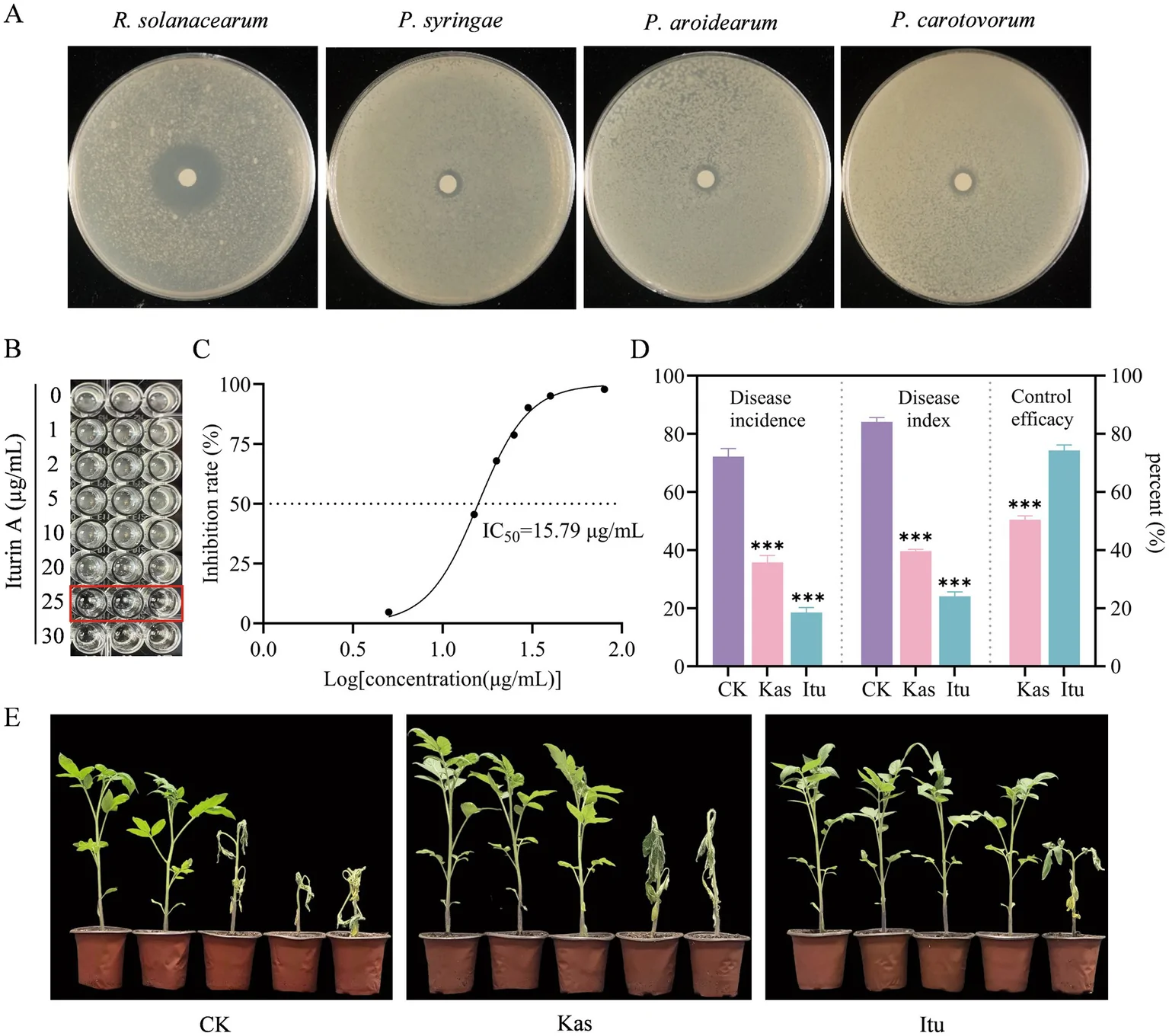
Detection of the antibacterial activity of Iturin A. **(A)** Antibacterial activity of Iturin A against four pathogenic bacteria. **(B)** MIC and **(C)** IC_50_ of Iturin A against *R. solanacearum* Cas_800_. **(D)** Disease incidence, disease index, and control efficacy of the control, kasugamycin (Kas), and Iturin A (Itu) groups in pot experiments. **(E)** Pot experiment results of control group, Kas, and Itu. *p < 0.05; **p < 0.01; ***p < 0.001.

Pot experiments further demonstrated the biocontrol efficacy of Iturin A against tomato bacterial wilt (Figure 2D). The disease incidence of the Iturin A-treated group (24.07 ± 0.93%) was significantly lower than that of CK (84.12 ± 0.88%) and the kasugamycin-treated (39.63 ± 0.37%) group (*p* < 0.05). Compared with CK, the disease index of the Iturin A-treated group decreased by 74.27%, whereas that of the kasugamycin-treated group decreased by 50.96%. Accordingly, the control efficacy of Iturin A was 1.45-fold higher than that of kasugamycin (Figures 2D,E).

### 3.2 Changes in *Ralstonia solanacearum* morphology and ultrastructure induced by Iturin A

The effects of Iturin A on the morphology and ultrastructure of *R. solanacearum* were examined by SEM and TEM (Figure 3). Untreated *R. solanacearum* cells exhibited typical plump, short-rod structures (Figure 3A). Treatment with 16 μg/mL Iturin A induced cell shrinkage, and progressively higher concentrations caused further structural damage and intercellular adhesion (Figure 3A). TEM observations revealed that untreated cells possessed intact cell membranes and well-defined intracellular structures, whereas treatment with 16 μg/mL Iturin A induced membrane invagination. At higher concentrations (25 μg/mL), membrane disruption progressed to rupture and cytoplasmic leakage (Figure 3B). Collectively, the SEM and TEM results indicate that Iturin A compromises the structural integrity of *R. solanacearum* cell membranes in a concentration-dependent manner.

**Figure 3:**
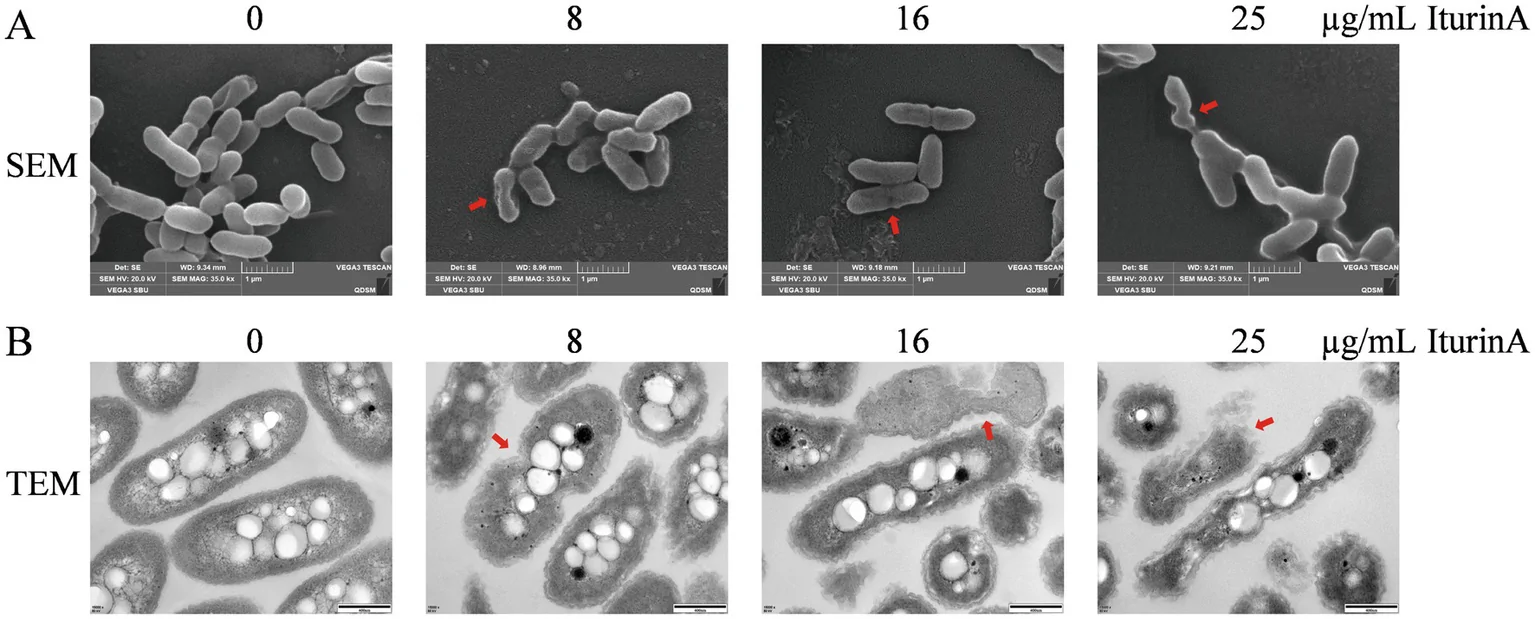
Changes in morphology and ultrastructure of *R. solanacearum* Cas_800_ induced by Iturin A. **(A)** SEM and **(B)** TEM images of *R. solanacearum* treated with 0 and 25 µg/mL Iturin A.

### 3.3 Effects of Iturin A on membrane integrity

To further assess the effect of Iturin A on *R. solanacearum* membrane integrity, a GFP expressing *R. solanacearum* strain was employed. This approach exploits the principle that GFP leaks through damaged membranes, resulting in diminished intracellular fluorescence. GFP fluorescence intensity decreased significantly after Iturin A treatment (Figure 4A), with reductions of 19.90, 36.10, and 74.21% at concentrations of 16, 25, and 50 μg/mL, respectively. These results confirmed that Iturin A disrupts *R. solanacearum* cell-membrane integrity in a dose-dependent manner, leading to cytoplasmic leakage.

**Figure 4:**
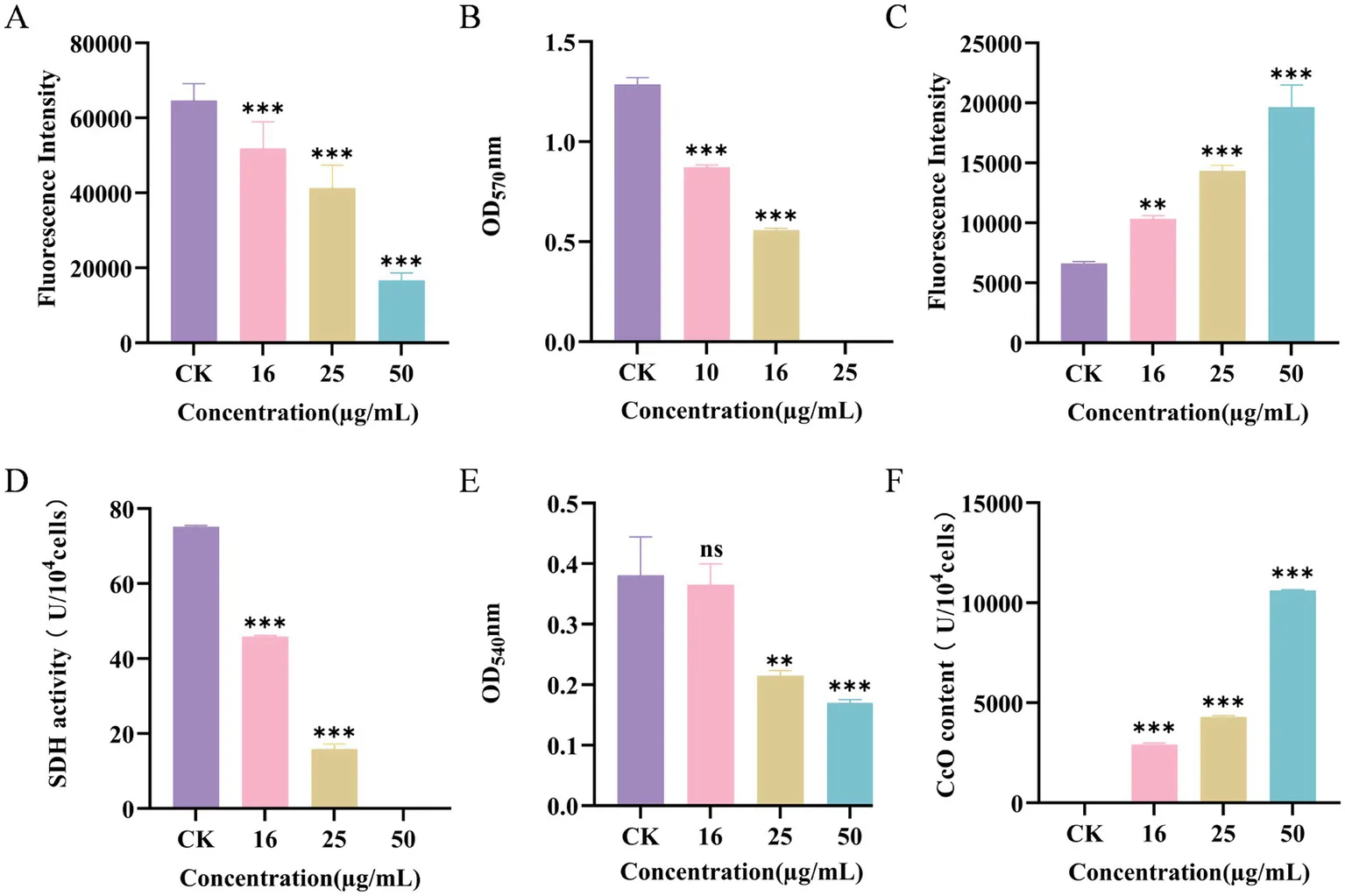
Iturin A-induced effect on membrane integrity, biofilm formation ability, oxidative stress, and electron transport of *R. solanacearum* Cas_800_. **(A)** Cell membrane integrity. **(B)** Biofilm biomass formation ability. **(C)** ROS levels. **(D)** SDH activity. **(E)** The total content of NAD^+^ and NADH. **(F)** CcO content. *p < 0.05; **p < 0.01; ***p < 0.001.

### 3.4 Effects of Iturin A on biofilm formation and ROS accumulation

Crystal-violet staining revealed that Iturin A significantly inhibited *R. solanacearum* biofilm formation in a concentration-dependent manner (*p* < 0.05; Figure 4B). At 10 μg/mL and 16 μg/mL, biofilm formation was reduced by 31.89 and 56.65%, respectively, whereas treatment at 25 μg/mL resulted in near-complete inhibition (Figure 4B). Intracellular ROS levels, assessed using the DCFH-DA fluorescent probe, increased significantly following Iturin A treatment in a concentration-dependent manner (*p* < 0.05; Figure 4C). ROS levels in *R. solanacearum* cultures treated with 16, 25, and 50 μg/mL Iturin A were 1.6-, 2.2-, and 3.0-fold higher, respectively, than those in the untreated control (Figure 4C).

### 3.5 Effects of Iturin A on the electron transport chain

SDH, a key enzyme linking the tricarboxylic acid (TCA) cycle and the ETC, was significantly inhibited by Iturin A in a concentration-dependent manner (*p* < 0.05; Figure 4D). Treatment with 16 and 25 μg/mL Iturin A reduced SDH activity to 38.99 and 78.84%, respectively, whereas activity was undetectable at 50 μg/mL. These results indicate that Iturin A impairs TCA cycle function and ETC activity in *R. solanacearum* through SDH inhibition.

Total NAD^+^ and NADH contents, reflecting the overall cellular metabolic status, were also significantly diminished following Iturin A treatment (Figure 4E). A modest reduction was observed at 16 μg/mL, whereas treatment at 25 and 50 μg/mL caused significant decreases of 43.65 and 55.35%, respectively (*p* < 0.05, Figure 4E).

CcO, the terminal oxidase of the ETC was assessed by DAB staining. Compared with the untreated control, the positively stained area indicating CcO in *R. solanacearum* exhibited a dose-dependent increase across all three concentrations of Iturin A (Figure 4F). The elevated CcO content suggests accumulation or aberrant localisation of terminal oxidase components resulting from impairment of upstream ETC processes, further corroborating that Iturin A disrupts the respiratory electron transport chain of *R. solanacearum*.

### 3.6 Transcript analysis of *Ralstonia solanacearum* response to Iturin A and qPCR validation

To elucidate the transcriptional response of *R. solanacearum* to Iturin A, RNA sequencing was performed on bacterial cultures treated at the IC₅₀ concentration (16 μg/mL). A high total mapping rate (95.18%) to the reference genome confirmed the quality and specificity of the transcriptome data (Supplementary Table S2). Of the total detected genes, 4,803 were co-expressed in both the treated and control groups, while 19 and 24 genes were uniquely expressed in Iturin A-treated and CK groups, respectively (Figure 5A). Differential expression analysis identified 168 upregulated and 336 downregulated DEGs (|log2FC| > 1, *p* < 0.05; Figure 5B). GO enrichment analysis revealed that DEGs were primarily associated with biological processes, including proteins and peptides biosynthesis and metabolism (Figure 5C). KEGG pathway analysis revealed significantly enrichment in ribosome function, secondary metabolite biosynthesis, amino acid biosynthesis, flagellar assembly, and oxidative phosphorylation (Figure 5D). Notable DEGs included *pilQ* in the flagellar assembly pathway; *galU,* shared by the starch and sucrose metabolism and O-antigen nucleotide sugar biosynthesis pathways; *sbnA*, *snbB*, *sbnB* in secondary metabolite biosynthesis; and *YscC*, *YscJ*, *popA*, *popB*, *secY*, and other genes in the bacterial secretion system pathway.

**Figure 5:**
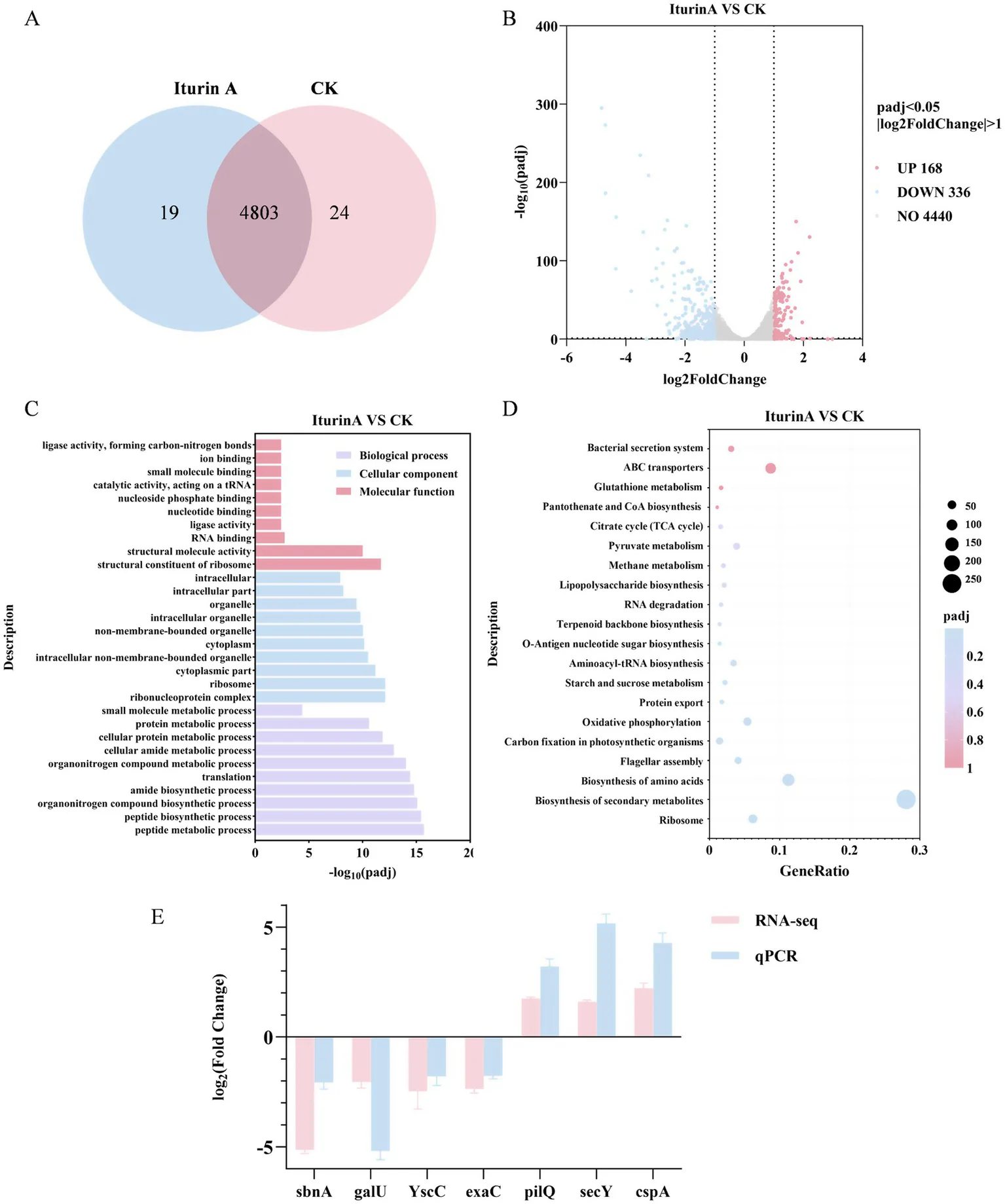
Transcript analysis and qPCR validation of *R. solanacearum* treated with Iturin A. **(A)** Venn diagram of genes expressed in the Iturin A and CK groups. **(B)** Volcano diagram of Iturin A and CK. **(C)** GO enrichment of functional annotation. **(D)** KEGG analysis of Iturin A vs. CK. **(E)** Expression changes of seven DEGs selected for validation of RNA-Seq data by qRT-PCR. qRT-PCR assays were performed in three individual experiments.

To validate the transcriptomic data, seven key DEGs were selected for qPCR analysis based on their physiological functions and relevance to *R. solanacearum* virulence. Among the downregulated genes, *galU*, encoding uridine diphosphate glucose pyrophosphorylase (UGP), was suppressed by 9.5-fold; *YscC*, encoding an outer-membrane ring channel protein of the T3SS, was downregulated 3.41-fold; *sbnA*, encoding a pyridoxal 5′-phosphate-dependent enzyme, was downregulated 36.3-fold; and *exaC*, encoding an aldehyde dehydrogenase, was downregulated 3.4-fold. Among the upregulated genes, *pilQ* (fimbrial assembly), *secY* (transmembrane transport), and *cspA* (cold shock protein) were increased 9.3-, 36.44-, and 28.93-fold, respectively (Figure 5E). These qPCR results were consistent with the transcriptomic data, confirming the reliability and accuracy of the RNA-seq analysis.

## 4 Discussion

*Ralstonia solanacearum* causes one of the most destructive bacterial wilt diseases in crops worldwide, and its control remains a significant challenge. Although chemical bactericides such as thiodiazole copper are currently employed as primary control measures, the emergence of pathogen resistance necessitates the development of alternative bio-derived antimicrobial agents. Our work demonstrated that Iturin A, derived from *B. amyloliquefaciens* Cas02, inhibits *R. solanacearum*; however, its control efficacy and mode of action remain unclear. In the study, we systematically evaluated the antibacterial activity and biocontrol efficacy of Iturin A against *R. solanacearum* through plate assays, 96-well plate assays, and pot experiments, and elucidated its mode of action through phenotypic assays and transcriptomic analysis. Our results demonstrate that Iturin A inhibits *R. solanacearum* growth at low concentrations (MIC = 25.00 μg/mL; IC₅₀ = 15.79 μg/mL) through multiple mechanisms, including cell-membrane disruption, biofilm-formation suppression, oxidative-stress induction, and ETC interference (Figure 6).

**Figure 6:**
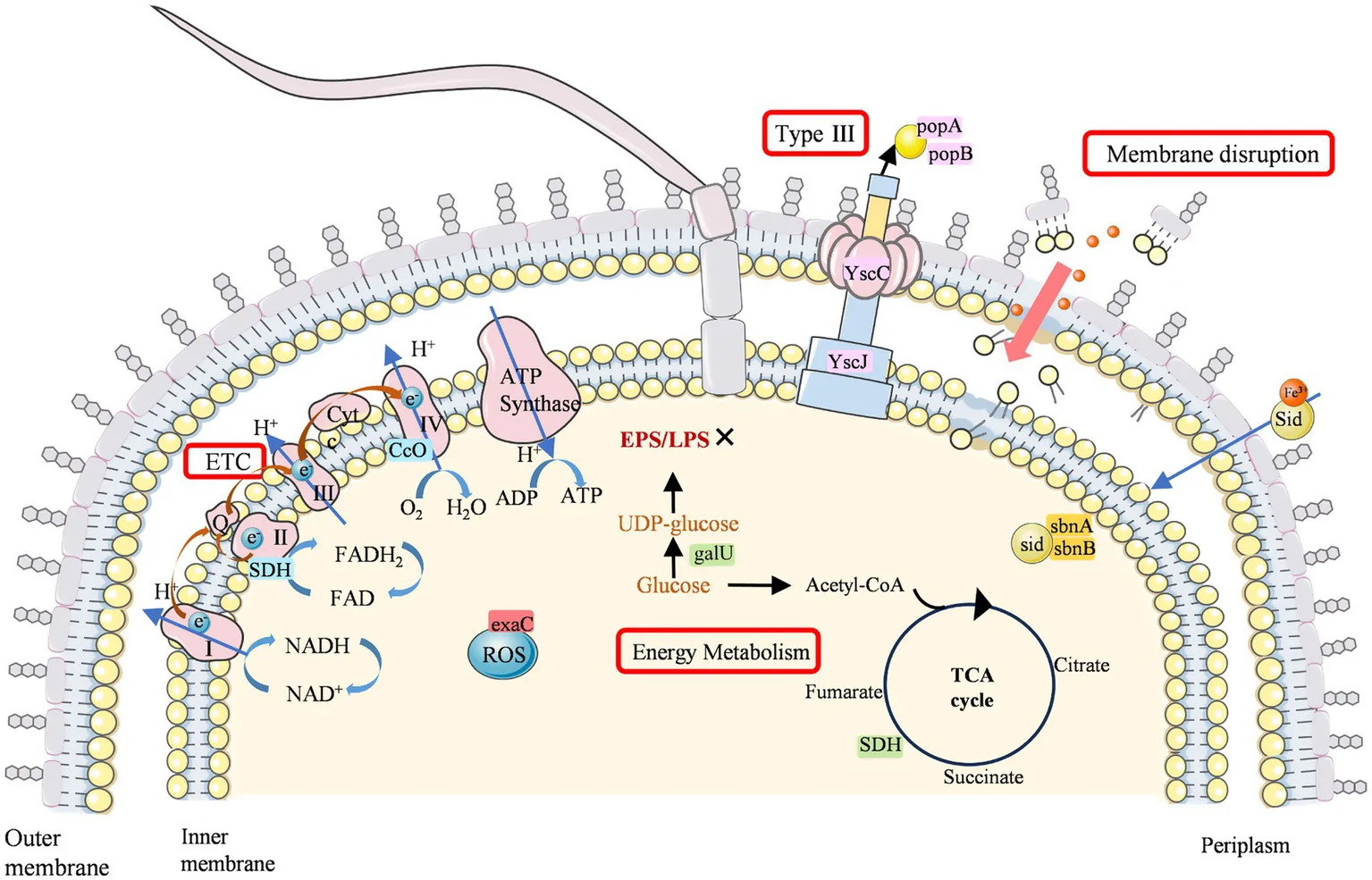
Schematic diagram of the mechanism of action of Iturin A against *R. solanacearum*.

Iturin A possesses broad application potential in agricultural biocontrol. Beyond its well-documented antifungal and insecticidal activities in agriculture and food preservation (Kumar et al., 2017; Masmoudi et al., 2023), Iturin A also exhibits antiviral and anti-proliferative properties of medical relevance (Jiang et al., 2021; Shekunov et al., 2023; Yadav and Eswari, 2022). However, reports on its efficacy against bacterial plant pathogens remain limited. Several studies have demonstrated that Iturin A exhibits pronounced antibacterial activity against *R. solanacearum*; the present study further dissects the underlying basis of this phenomenon. Notably, the MIC of Iturin A (25 μg/mL) was substantially lower than that of copper hydroxide (200 μg/mL) reported by Lee et al. (2012) for chemical fungicide combinations against *R. solanacearum*. Furthermore, pot experiments confirmed that the control efficacy of Iturin A against tomato bacterial wilt surpassed that of kasugamycin, highlighting its potential as an antibacterial agent. For bacterial wilt management.

The cell membrane of *R. solanacearum* consists of an inner membrane, a thin layer of peptidoglycan, and an outer membrane, which is essential for maintaining cellular homeostasis and mediating material exchange and constitutes a primary target of numerous antibiotics (Ntallis et al., 2025). SEM and TEM in this study revealed that Iturin A induced substantial structural damage to *R. solanacearum* cells: lower concentrations caused cell shrinkage, deformation, and membrane invagination, whereas higher concentrations led to membrane rupture and intracellular-content leakage. GFP leakage assay results further corroborated these observations, consistent with previous reports that the amphipathic molecular structure of Iturin A enables it to penetrate and perforate pathogen cell membranes (Ongena and Jacques, 2007; Wan et al., 2022). Although the disruptive effects of Iturin A on fungal cell membranes have been extensively documented (Arrebola et al., 2010), our ultrastructural analysis demonstrated that bacterial membranes are also highly susceptible to Iturin A-induced damage. Furthermore, biofilm formation represents a critical survival strategy for *R. solanacearum*, facilitating soil persistence, xylem colonisation, and enhanced tolerance to environmental stress and antimicrobial agents (Du et al., 2024; Lowe-Power et al., 2018). The present study demonstrated that Iturin A significantly inhibits *R. solanacearum* biofilm formation, consistent with the findings of Englerová et al. (2021), which found that Iturin A reduces *Staphylococcus aureus* adhesion and biofilm formation. Collectively, these results indicate that Iturin A exerts antibacterial effects against *R. solanacearum* in part through disruption of membrane structure and inhibition of biofilm formation.

Excessive ROS accumulation or an imbalance in the antioxidant system leads to oxidative stress, resulting in cellular damage and functional impairment (Iqbal et al., 2024; Jomova et al., 2023). In this study, DCFH-DA fluorescence intensity increased proportionally with Iturin A concentration, indicating that Iturin A induced dose-dependent oxidative stress in *R. solanacearum.* These findings are consistent with those of Zou et al. (2025), which demonstrated that membrane disruption by the biomimetic lipopeptide LP3K induced oxidative stress in both *S. aureus* and *Pseudomonas aeruginosa*. Thus, the antibacterial activity of Iturin A may be partially attributable to the induction of oxidative stress. Furthermore, Iturin A treatment led to a significant, concentration-dependent decrease in SDH activity, accompanied by marked reductions in NAD^+^ and NADH contents, indicating impairment of both the TCA cycle and the ETC. These results are consistent with the findings of Wang et al. (2024), who demonstrated that Iturin A significantly decreased SDH activity in *A. niger*, thereby interfering with the TCA cycle and impairing cellular respiratory metabolism. The enhanced CcO staining observed following Iturin A treatment suggests accumulation or abnormal localisation of terminal oxidase components under stress conditions, further corroborating ETC disruption. Taken together, these findings suggest that Iturin A inhibits *R. solanacearum* by disrupting energy metabolism and electron transport.

Transcriptomic analysis and qPCR validation provided additional insights into the molecular mechanisms underlying the antibacterial action of Iturin A. DEGs were enriched in pathways associated with ribosome function, amino acid biosynthesis, secondary metabolism, and flagellar assembly, indicating that protein synthesis, metabolic flux, and motility of *R. solanacearum* are extensively reprogrammed under subinhibitory concentrations of Iturin A. The *galU* gene encodes UGP, whose product, UDP-glucose (UDP-Glc), serves as a central precursor for the biosynthesis of lipopolysaccharides, capsular polysaccharides, and exopolysaccharides—key virulence determinants of *R. solanacearum* (Banerjee et al., 2025; Ramón Roth et al., 2025). Functional deficiency of *galU* directly compromises virulence factor biosynthesis and significantly attenuates the survival and pathogenicity of *R. solanacearum*, consistent with the observed reduction in biofilm formation. *YscC* and *YscJ* encode essential structural components of the T3SS: *YscC* encodes an outer-membrane ring channel protein (Kowal et al., 2013), and *YscJ* encodes a key periplasmic-bridging protein that maintains the structural continuity of the secretion apparatus (Tampakaki et al., 2004). Downregulation of these genes would impair T3SS assembly and abrogate effector protein secretion, thereby attenuating *R. solanacearum* virulence. Similarly, the T3SS effector proteins *popA* and *popB*, which act as elicitors inducing the hypersensitive response in host plants (Van Gijsegem et al., 2000), were significantly downregulated. The gene of *exaC* encodes an aldehyde dehydrogenase catalysing the detoxification of acetaldehyde during ethanol metabolism (Ko et al., 2025); its downregulation may lead to acetaldehyde accumulation, promoting ROS generation and oxidative damage, consistent with the elevated intracellular ROS levels observed in this study. The significant downregulation of siderophore biosynthesis genes (*sbnA* and *sbnB*) would impair iron acquisition, a process critical for bacterial growth, survival, and virulence (Dale et al., 2004; Schalk, 2025). Conversely, the upregulation of *pilQ* and *secY* may represent compensatory responses to maintain protein secretion and surface structures under stress (Blattman et al., 2024), while increased *cspA* expression reflects activation of a general stress response (Smith et al., 2023). Collectively, these results demonstrate that Iturin A not only exerts bactericidal effects on *R. solanacearum* but also attenuates its virulence by modulating multiple pathways associated with pathogenicity and environmental adaptation, providing a mechanistic explanation for its high biocontrol efficacy.

In summary, through integrated *in vivo* and *in vitro* assays, physiological and biochemical analyses, and transcriptomic profiling, this study elucidates a multitargeted mode of action by which Iturin A inhibits *R. solanacearum*: disruption of cell-membrane structure and biofilm formation, interference with the ETC and energy metabolism, induction of ROS accumulation, and downregulation of multiple virulence-associated genes. This synergistic mode of action effectively suppresses tomato bacterial wilt and may reduce the likelihood of resistance development.

Several limitations of this study should be acknowledged. First, some experiments were conducted *in vitro* or under controlled pot conditions, therefore multi-season field trials are required to validate the efficacy of Iturin A against bacterial wilt under natural conditions. Moreover, given that bacterial wilt is difficult to cure once symptoms develop and that prophylactic application therefore remains the primary control strategy, the present pot experiment assessed only the protective efficacy of Iturin A applied prior to pathogen inoculation. Future studies will evaluate the curative efficacy (drug application after *R. solanacearum* inoculation) of Iturin A under both pot and field conditions. Finally, limitations in fermentation technology remain a bottleneck constraining the development of microbial pesticides. Optimizing the fermentation process for Iturin A and reducing its production cost are prerequisites for its practical application.

## 5 Conclusion

This study provides insights into the multitargeted antibacterial mechanisms of Iturin A against *R. solanacearum*. Iturin A significantly inhibited the growth of *R. solanacearum*, with an MIC of 25.00 μg/mL and an IC₅₀ of 15.79 μg/mL. Mechanistic investigations demonstrated that Iturin A exerts direct antibacterial effects primarily through disruption of cell-membrane integrity, inhibition of biofilm formation, induction of oxidative stress, and impairment of energy metabolism and electron transport. Transcriptomic and qPCR analyses revealed that Iturin A downregulates the following genes: those associated with cell-membrane and biofilm biosynthesis (*galU*), the T3SS (*YscC*, *YscJ*, *popA*, and *popB*), ROS detoxification (*exaC*), and siderophore biosynthesis (*sbnA* and *sbnB*). Pot experiments confirmed that Iturin A provides superior biocontrol efficacy against tomato bacterial wilt compared with kasugamycin. These findings collectively indicate that Iturin A suppresses *R. solanacearum* through a synergistic mode of action involving direct membrane damage, metabolic disruption, and attenuation of multiple virulence pathways, supporting its potential as a natural bactericide for the management of tomato bacterial wilt.

## Data Availability

The database information and accession number for the transcriptome data have been added to the Supplementary materials.
