# Supplementary material for: Multitargeted antibacterial mechanisms of Iturin A derived from Bacillus amyloliquefaciens against tomato bacterial wilt
Source: Front Microbiol. 2026 Oct 20;17:1944894. doi: 10.3389/fmicb.2026.1944894 (PMC13728855; doi:10.3389/fmicb.2026.1944894)
Supplement: Supplementary file 1 [file Table_1.docx]

Supplementary Material

# 1 Supplementary Tables

**Table S1 Primers used for qRT-PCR in this study**

| Gene ID | Gene name | Primer sequences (5′ to 3′) |
| --- | --- | --- |
| RS_RS19170 | sbnA | F: ACCAACCTGAAGATCCTGCG |
|  |  | R: TACTGGTTGATCCACACCGC |
| RS_RS17225 | galU | F: CGAGCCCTACGCCGACAAC |
|  |  | R: GACCAGCAGCGGCACCAG |
| RS_RS21315 | YscC | F: CAGAACTTCTACGTGCCGGT |
|  |  | R: TGATCTGGCCGTCTTCGATG |
| RS_RS15715 | exaC | F: ATCCGCACCTACCTCGAGAT |
|  |  | R: CCTGGAACACGCGCATATTG |
| RS_RS14890 | pilQ | F: CAGTGAGCTTGTTCAACGCC |
|  |  | R: CGATCAGCGCCTTGATGTTG |
| RS_RS15025 | SecY | F: CAGCTTTTCCAACGGCAGTC |
|  |  | R: CAAATACCGTGAAGCGCGAG |
| RS_RS15860 | cspA | F: TTTGGCTTCATCACGCCTGA |
|  |  | R: TCTTGCAGCGTTTTGAAGCC |

**Table S2** **Statistics of reads mapping to the reference genome in control and treatment groups**

| Sample_name | CK1 | CK2 | CK3 | T1 | T2 | T3 |
| --- | --- | --- | --- | --- | --- | --- |
| Total reads | 14534594 | 14457596 | 12846956 | 15117290 | 14494548 | 15152156 |
| Total mapped reads | 13913990 | 13778893 | 12228145 | 14494701 | 13964715 | 14558024 |
| Uniquely mapped reads | 13247258 | 13087841 | 11552425 | 13792941 | 13335628 | 13889780 |
| Multiple mapped reads | 666732 | 691052 | 675720 | 701760 | 629087 | 668244 |
| Total mapping rate | 95.73% | 95.31% | 95.18% | 95.88% | 96.34% | 96.08% |
| Uniquely mapping rate | 91.14% | 90.53% | 89.92% | 91.24% | 92% | 91.67% |
| Multiple mapping rate | 4.59% | 4.78% | 5.26% | 4.64% | 4.34% | 4.41% |

# 2 Data availability statement

The transcriptome data were deposited in the NCBI Sequence Read Archive (SRA) database

402 under the accession number PRJNA1415523.
